# The WOEST 2 registry: A prospective registry on antithrombotic therapy in atrial fibrillation patients undergoing percutaneous coronary intervention

**DOI:** 10.1007/s12471-022-01664-0

**Published:** 2022-03-01

**Authors:** A. J. W. M. de Veer, N. Bennaghmouch, W. L. Bor, J. P. R. Herrman, M. Vrolix, M. Meuwissen, T. Vandendriessche, T. Adriaenssens, B. de Bruyne, M. Magro, W. J. M Dewilde, J. M. ten Berg

**Affiliations:** 1 Department of Cardiology, St. Antonius Hospital, Nieuwegein, The Netherlands; 2 Department of Cardiology, OLVG, Amsterdam, The Netherlands; 3 Department of Cardiology, Ziekenhuis Oost-Limburg, Genk, Belgium; 4 Department of Cardiology, Amphia Hospital, Breda, The Netherlands; 5 Department of Cardiology, Antwerp University Hospital, Edegem, Belgium; 6 Department of Cardiovascular Medicine, University Hospital Leuven, Leuven, Belgium; 7 Cardiovascular Research Centre Aalst, OLV Clinic, Aalst, Belgium; 8 Department of Cardiology, Elizabeth-TweeSteden Hospital, Tilburg, The Netherlands; 9 Department of Cardiology, Imelda Hospital, Bonheiden, Belgium

**Keywords:** Anticoagulation, Antiplatelet therapy, Acute coronary syndrome, Percutaneous coronary intervention, Atrial fibrillation, Non-vitamin, Oral anticoagulants

## Abstract

**Background:**

Patients on oral anticoagulants (OACs) undergoing percutaneous coronary intervention (PCI) also require aspirin and a P2Y12 inhibitor (triple therapy). However, triple therapy increases bleeding. The use of non-vitamin K antagonist oral anticoagulants (NOACs) and stronger P2Y12 inhibitors has increased. The aim of our study was to gain insight into antithrombotic management over time.

**Methods:**

A prospective cohort study of patients on OACs for atrial fibrillation or a mechanical heart valve undergoing PCI was performed. Thrombotic outcomes were myocardial infarction, stroke, target-vessel revascularisation and all-cause mortality. Bleeding outcome was any bleeding. We report the 30-day outcome.

**Results:**

The mean age of the 758 patients was 73.5 ± 8.2 years. The CHA_2_DS_2_-VASc score was ≥ 3 in 82% and the HAS-BLED score ≥ 3 in 44%. At discharge, 47% were on vitamin K antagonists (VKAs), 52% on NOACs, 43% on triple therapy and 54% on dual therapy. Treatment with a NOAC plus clopidogrel increased from 14% in 2014 to 67% in 2019. The rate of thrombotic (4.5% vs 2.0%, *p* = 0.06) and bleeding (17% vs. 14%, *p* = 0.42) events was not significantly different in patients on VKAs versus NOACs. Also, the rate of thrombotic (2.9% vs 3.4%, *p* = 0.83) and bleeding (18% vs 14%, *p* = 0.26) events did not differ significantly between patients on triple versus dual therapy.

**Conclusions:**

Patients on combined oral anticoagulation and antiplatelet therapy undergoing PCI are elderly and have both a high bleeding and ischaemic risk. Over time, a NOAC plus clopidogrel became the preferred treatment. The rate of thrombotic and bleeding events was not significantly different between patients on triple or dual therapy or between those on VKAs versus NOACs.

**Supplementary Information:**

The online version of this article (10.1007/s12471-022-01664-0) contains supplementary material, which is available to authorized users.

## What’s new?

- Physicians are capable of choosing the right combination of drugs for patients on oral anticoagulation therapy undergoing percutaneous coronary intervention, e.g. triple therapy where a high thrombotic risk prevails and dual therapy for those with a high bleeding risk.
- The thrombotic and bleeding event rates were not significantly different in patients on triple versus dual therapy, nor in those on vitamin K antagonists versus non-vitamin K antagonist oral anticoagulants (NOACs).
- Over time, a NOAC plus clopidogrel has become the preferred treatment.

## Introduction

Chronic oral anticoagulant (OAC) therapy (class I) is recommended in patients with atrial fibrillation (AF) and a CHA_2_DS_2_-VASc score of ≥ 1 for men and ≥ 2 for woman [1], as well as for patients with a mechanical heart valve. When these patients undergo percutaneous coronary intervention (PCI) with stenting or suffer from acute coronary syndrome (ACS), dual antiplatelet treatment (DAPT) with aspirin and a P2Y12 inhibitor, such as clopidogrel, ticagrelor or prasugrel, is also indicated [2–4]. This so-called triple therapy (an OAC combined with DAPT) aims to minimise the risk of stroke and coronary ischaemic events [5]. However, triple therapy increases the risk for bleeding 2‑ to 3‑fold, and bleeding is associated with mortality [6]. Therefore, safer antithrombotic regimens are needed for these patients.

In the ISAR-TRIPLE trial, shortening the course of triple therapy was advocated as a strategy to reduce bleeding. Regrettably, 6 weeks of triple therapy did not reduce the risk of bleeding as compared to 6 months [7].

Reducing bleeding events by omitting aspirin has also been proposed. The WOEST (What is the Optimal antiplatElet and anticoagulant therapy in patients with oral anticoagulation undergoing revasculariSaTion) study and a real-life nationwide Danish registry of more than 12,000 patients demonstrated that omitting aspirin after PCI or ACS led to significantly less bleeding while not increasing the occurrence of ischaemic events [8, 9]. However, at that time only clopidogrel and vitamin K antagonists (VKAs) were available, while nowadays for ACS patients stronger P2Y12 inhibitors (prasugrel, ticagrelor) [10, 11] and for AF patients non-vitamin K oral anticoagulants (NOACs) are accessible [12–15].

In the absence of evidence from randomised controlled trials (RCTs) about the optimal antithrombotic strategy in patients with AF undergoing PCI, until 2014 clinicians had to rely on expert opinion such as position papers [16]. Since then, new evidence has come from RCTs (including NOACs and stronger P2Y12 inhibitors), subsequently leading to the preferred use of NOACs for the management of AF [1] and ticagrelor/prasugrel for ACS [3, 4]. Nevertheless, there seems to be large variance between countries, but also between centres, regarding the antithrombotic therapy used in these patients.

To gain insight into what cardiologists prefer as optimal antithrombotic treatment in patients with AF undergoing PCI, we conducted the WOEST 2 registry study in two countries. The main goal of the WOEST 2 Registry was to improve medical care for patients with AF undergoing PCI through a better understanding of their demographics, antithrombotic management and related outcomes.

### Methods

The WOEST 2 Registry is an international, multi-centre, non-interventional, cohort study in patients on OACs (for AF or a mechanical heart valve) undergoing PCI.

Between March 2014 and July 2019, we identified patients at nine sites in the Netherlands and Belgium. We included patients requiring oral anticoagulation for AF or a mechanical heart valve undergoing PCI (elective or urgent). We collected information on antithrombotic therapy prior to, during and after PCI. All decisions regarding the treatment of patients were made by the treating physician. After signing the informed consent form, patients were included in the registry. Follow-up was 30 days. The thrombotic outcome was the composite of myocardial infarction, ischaemic stroke (including transient ischaemic attack), target-vessel revascularisation and all-cause mortality. The bleeding outcome was the incidence of any bleeding. Descriptive analysis of the data was performed using summary statistics for categorical and quantitative (continuous) data. Continuous data are reported as means with standard deviation or medians with interquartile range. Categorical data are expressed as percentages. Distributions of categorical data were examined by Fisher’s exact test. Continuous data were compared using Student’s *t*-test or the Mann-Whitney U test, as appropriate. Predictors for different treatment regimens or outcomes were identified by logistic regression. For comparisons between VKAs and NOACs, patients with mechanical heart valves and patients with an estimated glomerular filtration rate (eGFR) < 30 were excluded, since they are not eligible for (all) NOACs. The analyses were performed using RStudio for Windows, version 1.2.

Due to the non-interventional character of the registry the ethics committee decided that the Medical Research Involving Human Subjects Act (WMO) did not apply to this registry and therefore official approval by the ethics committee was not required. Declaration of approval of the Institutional Review Board (LMTE) was obtained. A local study protocol is available. The study was conducted in accordance with the Declaration of Helsinki.

### Results

A total of 758 patients were included from March 2014 to July 2019. Their baseline characteristics are shown in Tab. 1. Of all patients, 8% used OACs for a mechanical heart valve prosthesis, the remainder (92%) for AF. Mean age was 73.5 ± 8.2 years, and 25% were female. Prior coronary artery disease was present in 74%, congestive heart failure in 23% and valvular disease in 21% of the patients. For 14% of the patients previous stroke/transient ischaemic attack and for 11% previous bleeding was reported. The CHA_2_DS_2_-VASc score was ≥ 3 in 82% of patients and the HAS-BLED score ≥ 3 in 44% of patients. Antithrombotic therapy before hospital admission consisted of aspirin in 21%, P2Y12 inhibitors in 29% and OACs in 89% of the patients. In 9%, AF was newly diagnosed.

**Table 1:** Baseline characteristics

	Overall (*n* = 758)	NOAC (*n* = 393)	VKA (*n* = 353)	*p*-value	DT (*n* = 437)	TT (*n* = 309)	*p*-value
*Comorbidities*							
Age (mean (SD))	73.58 (8.24)	73.67 (8.07)	73.36 (8.45)	0.610	73.58 (8.33)	73.44 (8.14)	0.819
– > 75 years (%)	355 (46.8)	188 (47.8)	160 (45.3)	0.509	206 (47.1)	142 (46.0)	0.766
Female (%)	186 (24.5)	107 (27.2)	77 (21.8)	0.090	111 (25.4)	73 (23.6)	0.606
Caucasian ethnicity (%)	716 (94.5)	369 (93.9)	336 (95.2)	0.521	415 (95.0)	290 (93.9)	0.518
Smoker (%)	110 (14.8)	61 (15.6)	47 (13.7)	0.531	61 (14.2)	47 (15.6)	0.598
Alcohol abuse > 7 units/week (%)	75 (11.4)	37 (10.2)	38 (13.2)	0.266	60 (16.1)	15 (5.4)	< 0.001
Diabetes mellitus (%)	208 (27.4)	104 (26.5)	102 (28.9)	0.462	124 (28.4)	82 (26.5)	0.618
Atrial fibrillation (%)	695 (91.7)	382 (97.2)	302 (85.6)	< 0.001	402 (92.0)	282 (91.3)	0.788
*Type of atrial fibrillation*							
New-onset/recently diagnosed	61 (8.9)	39 (10.5)	21 (7.0)		36 (9.1)	24 (8.7)	
Paroxysmal	327 (47.9)	193 (51.7)	128 (43.0)		188 (47.5)	133 (48.4)	
Persistent	62 (9.1)	46 (12.3)	16 (5.4)		33 (8.3)	29 (10.5)	
Long-standing persistent	61 (8.9)	28 (7.5)	32 (10.7)		38 (9.6)	22 (8.0)	
Permanent	171 (25.1)	67 (18.0)	101 (33.9)		101 (25.5)	67 (24.4)	
Prior CAD (%)	561 (74.0)	275 (70.0)	279 (79.0)	0.006	331 (75.7)	223 (72.2)	0.308
Prior MI (%)	215 (28.4)	90 (22.9)	121 (34.4)	0.001	134 (30.7)	77 (25.0)	0.099
Prior PCI (%)	272 (35.9)	126 (32.1)	144 (40.8)	0.015	169 (38.7)	101 (32.7)	0.104
Prior CABG (%)	150 (19.8)	59 (15.0)	89 (25.2)	0.001	91 (20.8)	57 (18.4)	0.456
Congestive heart failure (%)	174 (23.0)	73 (18.6)	96 (27.4)	0.005	120 (27.5)	49 (16.0)	< 0.001
Valvular disease (%)	162 (21.4)	66 (16.8)	93 (26.3)	0.002	110 (25.2)	49 (15.9)	0.003
Valve surgery (%)	62 (8.2)	10 (2.5)	51 (14.4)	< 0.001	39 (8.9)	22 (7.1)	0.417
Prior stroke/TIA (%)	106 (14.0)	46 (11.7)	58 (16.4)	0.072	67 (15.3)	37 (12.0)	0.200
Prior PAD (%)	119 (15.7)	57 (14.5)	60 (17.0)	0.365	70 (16.0)	47 (15.2)	0.838
Prior bleeding (requiring medical attention) (%)	81 (10.7)	36 (9.2)	43 (12.2)	0.191	55 (12.6)	24 (7.8)	0.040
Prior GI bleeding (%)	28 (3.7)	14 (3.6)	13 (3.7)	1.000	18 (4.1)	9 (2.9)	0.432
Anaemia (%)	68 (9.0)	30 (7.6)	37 (10.5)	0.200	49 (11.2)	18 (5.8)	0.013
Chronic renal insufficiency (%)	158 (20.8)	85 (21.6)	71 (20.1)	0.652	101 (23.1)	55 (17.8)	0.083
*Present or prior malignancy (%)*	94 (12.4)	46 (11.7)	43 (12.2)	0.910	62 (14.2)	27 (8.7)	0.029
CHA_2_DS_2_-VASc (mean (SD))	3.94 (1.60)	3.84 (1.54)	4.06 (1.66)	0.065	4.11 (1.58)	3.70 (1.61)	0.001
CHA_2_DS_2_-VASc ≥ 3 (%)	619 (81.7)	320 (81.4)	290 (82.2)	0.849	373 (85.4)	237 (76.7)	0.003
HAS-BLED (median (IQR))	2.48 (1.18)	2.36 (1.16)	2.60 (1.20)	0.005	2.47 (1.04)	2.48 (1.37)	0.941
HAS-BLED ≥ 3 (%)	334 (44.2)	141 (36.0)	188 (53.3)	< 0.001	202 (46.3)	127 (41.1)	0.178
*Prior medication*							
Aspirin (%)	160 (21.1)	101 (25.7)	53 (15.0)	< 0.001	62 (14.2)	92 (29.8)	< 0.001
P2Y12 inhibitor (%)	217 (28.6)	112 (28.5)	102 (28.9)	0.935	126 (28.8)	88 (28.5)	0.935
VKA (%)	333 (43.9)	11 (2.8)	317 (89.8)	< 0.001	214 (49.0)	114 (36.9)	0.001
NOAC (%)	341 (45.0)	327 (83.2)	10 (2.8)	< 0.001	186 (42.6)	151 (48.9)	0.100

*NOAC* non-vitamin K antagonist, *VKA* vitamin K antagonist, *DT* dual therapy, *TT* triple therapy, *CAD* coronary artery disease, *MI* myocardial infarction, *PCI* percutaneous coronary intervention, *CABG* coronary artery bypass grafting, *TIA* transient ischaemic attack, *PAD* peripheral artery disease, *GI* gastro-intestinal

In-hospital data are shown in Tab. 2. Most PCIs (69%) were performed in an elective setting and in 31% for ACS. Of patients presenting with ACS, 22% underwent PCI for an ST-elevation myocardial infarction (STEMI), 61% for non-STEMI and 18% for unstable angina. In two-thirds of all patients, a radial access site was used. Drug-eluting stents (DES) were used in 93% of all patients. OACs were continued during PCI in an almost similar proportion of patients on VKAs (70%) and NOACs (65%). Additional unfractionated heparin (UFH) during PCI was used more frequently in patients on VKAs than for those on NOACs (94% vs 77%, *p* < 0.001). UFH was used in 85% of patients with a median dose of 7500 units. Bail-out glycoprotein IIb/IIIa inhibition was used in only 4% of patients. No bivalirudin use was reported.

**Table 2:** In-hospital data

	Overall (*n* = 758)	NOAC (*n* = 393)	VKA (*n* = 353)	*p*-value	DT (*n* = 437)	TT (*n* = 309)	*p*-value
*Admission*							
Acute coronary syndrome (%)	235 (31.0)	117 (29.8)	115 (32.6)	0.429	119 (27.2)	113 (36.6)	0.008
*ACS type*				0.113			0.079
Unstable AP	41 (17.4)	24 (20.5)	17 (14.8)		24 (20.2)	17 (15.0)	
NSTEMI	143 (60.9)	74 (63.2)	67 (58.3)		64 (53.8)	77 (68.1)	
STEMI	51 (21.7)	19 (16.2)	31 (27.0)		31 (26.1)	19 (16.8)	
LVEF < 50 (%)	0.35 (0.48)	0.32 (0.47)	0.39 (0.49)	0.131	0.40 (0.49)	0.26 (0.44)	0.001
Haemoglobin (median (IQR))	8.6 (7.8, 9.2)	8.7 (7.8, 9.3)	8.5 (7.8, 9.1)	0.094	8.6 (7.8, 9.2)	8.6 (7.9, 9.2)	0.801
Creatinine (median (IQR))	91 (78, 110)	90 (77, 107)	93 (79, 113)	0.082	91 (78, 111)	94 (78, 108)	0.835
eGFR	61 (49, 76)	63 (51, 77)	61 (48, 74)	0.074	61 (49, 75)	61 (50, 78)	0.659
*Procedure*							
Femoral access (%)	262 (34.6)	137 (34.9)	118 (33.4)	0.700	145 (33.2)	110 (35.6)	0.531
Interruption of OAC (%)	243 (33.1)	137 (35.2)	100 (29.9)	0.153	111 (26.4)	126 (41.7)	< 0.001
INR (median (IQR))	1.80 (1.30, 2.30)	1.20 (1.09, 1.48)	1.80 (1.40, 2.40)	< 0.001	1.90 (1.50, 2.40)	1.50 (1.20, 2.20)	< 0.001
Multivessel disease (%)	349 (46.0)	175 (44.5)	169 (47.9)	0.378	206 (47.1)	138 (44.7)	0.551
Bare-metal stent (%)	0.06 (0.24)	0.08 (0.27)	0.03 (0.18)	0.012	0.03 (0.17)	0.09 (0.29)	< 0.001
Drug-eluting stent (%)	706 (93.1)	364 (92.6)	333 (94.3)	0.377	417 (95.4)	280 (90.6)	0.011
LMWH use (including fondaparinux) (%)	0.14 (0.35)	0.20 (0.40)	0.07 (0.26)	< 0.001	0.06 (0.23)	0.25 (0.44)	< 0.001
Unfractionated heparin use (%)	642 (84.7)	302 (76.8)	331 (93.8)	< 0.001	405 (92.7)	228 (73.8)	< 0.001
Unfractionated heparin dose (median (IQR))	7500 (5000, 10.000)	7500 (5500, 10.000)	7500 (5000, 10.000)	0.559	7500 (6000, 10.000)	7500 (5000, 9000)	0.003
Glycoprotein IIb/IIIa antagonist use (%)	27 (3.6)	7 (1.8)	20 (5.7)	0.005	13 (3.0)	14 (4.5)	0.320
*Discharge medication*							
Aspirin (%)	319 (42.2)	184 (46.8)	128 (36.3)	0.004	3 (0.7)	309 (100.0)	< 0.001
P2Y12 inhibition (%)	753 (99.6)	390 (99.2)	353 (100.0)	0.251	434 (99.3)	309 (100.0)	0.271
Clopidogrel (%)	0.94 (0.25)	0.94 (0.24)	0.93 (0.25)	0.701	0.93 (0.26)	0.95 (0.22)	0.240
Ticagrelor (%)	0.06 (0.24)	0.05 (0.23)	0.07 (0.25)	0.498	0.07 (0.25)	0.05 (0.22)	0.310
Prasugrel (%)	0.00 (0.04)	0.00 (0.00)	0.00 (0.05)	0.292	0.00 (0.00)	0.00 (0.06)	0.235
OAC (%)	746 (98.7)	393 (100.0)	353 (100.0)	1.000	437 (100.0)	309 (100.0)	1.000
NOAC (%)	393 (52.0)	393 (100.0)	0 (0.0)	< 0.001	212 (48.5)	181 (58.6)	0.007
VKA (%)	353 (46.7)	0 (0.0)	353 (100.0)	< 0.001	225 (51.5)	128 (41.4)	0.007

*NOAC* non-vitamin K antagonist oral anticoagulant, *VKA* vitamin K antagonist, *DT* dual therapy, *TT* triple therapy, *ACS* acute coronary syndrome, *AP* angina pectoris, *NSTEMI* non-ST-elevation myocardial infarction, *STEMI* ST-elevation myocardial infarction, *LVEF* left ventricular ejection fraction, *IQR* interquartile range, *eGFR* estimated glomerular filtration rate, *OAC* oral anticoagulant, *INR* international normalised ratio, *LMWH* low-molecular-weight heparin

**Fig. 1:**
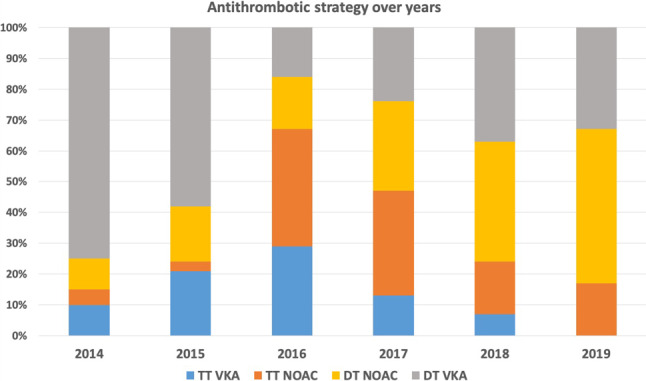
Antithrombotic strategy at discharge over time. *TT* triple therapy, *VKA* vitamin K antagonist, *NOAC* non-vitamin K oral anticoagulant, *DT* dual therapy

Switching among OACs during hospitalisation was not common: 11 patients switched from VKAs to NOACs and 13 patients from NOACs to VKAs. At discharge, 52% of the patients received NOACs, 47% received VKAs and only 3% received low-molecular-weight heparin. Triple therapy was prescribed to 43%, while 54% were on dual therapy consisting of a (N)OAC plus aspirin or a P2Y12 inhibitor. The P2Y12 inhibitor of choice was clopidogrel in 94% and ticagrelor in 6% of the patients. Less than 1% received prasugrel. Over the years, a strong trend towards more NOACs (14% in 2014 to 67% in 2019) and less triple therapy (62% in 2016 to 17% in 2019) was seen. Fig. 1 provides an overview of the prescription of OACs and antiplatelet therapy over time.

#### Patients on VKAs versus NOACs

Patients discharged on VKAs or NOACs were comparable in age, sex and body mass index. However, previous coronary artery disease, myocardial infarction, coronary artery bypass graft (CABG), congestive heart failure and valvular disease were more frequent in patients on VKAs than on NOACs (respectively 79% vs 70%, *p* < 0.01; 34% vs 23%, *p* < 0.01; 25% vs 15%, *p* < 0.01; 27% vs 19%, *p* < 0.01; 26% vs 17%, *p* < 0.01). A higher mean CHA_2_DS_2_-VASc score (4.0 vs 3.8) was found among patients on VKAs than in those on NOACs. Patients on VKAs received significantly less aspirin at discharge compared to those on NOACs (36% vs 47%, *p* < 0.01).

After correction for the year of the procedure, we found that the strongest predictors for VKA prescription were prior myocardial infarction, CABG, PCI, congestive heart failure, peripheral artery disease and STEMI at presentation (Electronic Supplementary Material, Table S1a).

#### Patients discharged on dual versus triple therapy

Patients discharged on dual therapy were comparable with those discharged on triple therapy with regard to age, sex, OAC indication, history of stroke, peripheral artery disease, renal failure and prior bleeding. Congestive heart failure (28% vs 16%, *p* < 0.01), valvular disease (25% vs 16%, *p* > 0.01), anaemia (11% vs 6%, *p* = 0.01) and malignancy (14% vs 9%, *p* = 0.03) were more frequent in patients discharged on dual therapy. In the triple therapy group, more ACS at presentation (37% vs 27%, *p* < 0.01) was found compared to patients discharged on dual therapy.

Significant predictors of triple therapy were prior hypertension, ACS at presentation and bare-metal stent placement (Electronic Supplementary Material, Table S1b). Congestive heart failure, valvular disease, malignancy and anaemia, or a higher CHA_2_DS_2_-VASc score were predictors for dual therapy.

#### Clinical outcomes

The clinical outcomes can be found in Tab. 3.

**Table 3:** Outcomes at 30 days

	Overall (*n* = 758)	NOAC (*n* = 393)	VKA (*n* = 353)	*p*-value	DT (*n* = 437)	TT (*n* = 309)	*p*-value
Bleeding (%)	119 (15.7)	66 (16.8)	51 (14.4)	0.420	63 (14.4)	54 (17.5)	0.263
*BARC*				0.237			0.599
1	22 (18.5)	12 (18.2)	10 (19.6)		13 (20.6)	9 (16.7)	
2	74 (62.2)	38 (57.6)	35 (68.6)		37 (58.7)	36 (66.7)	
3a	11 (9.2)	7 (10.6)	3 (5.9)		7 (11.1)	3 (5.6)	
3b	11 (9.2)	9 (13.6)	2 (3.9)		5 (7.9)	6 (11.1)	
3c	1 (0.8)	0 (0.0)	1 (2.0)		1 (1.6)	0 (0.0)	
4	0	0	0		0	0	
5	0	0	0		0	0	
*TIMI*				0.380			0.934
Major	6 (5.0)	5 (7.6)	1 (2.0)		3 (4.8)	3 (5.6)	
Minor	92 (77.3)	50 (75.8)	40 (78.4)		48 (76.2)	42 (77.8)	
Minimal	21 (17.6)	11 (16.7)	10 (19.6)		12 (19.0)	9 (16.7)	
*ISTH*				0.174			0.653
Major	23 (19.3)	16 (24.2)	6 (11.8)		13 (20.6)	9 (16.7)	
CRNM	76 (63.9)	38 (57.6)	37 (72.5)		38 (60.3)	37 (68.5)	
Minor	20 (16.8)	12 (18.2)	8 (15.7)		12 (19.0)	8 (14.8)	
All-cause death (%)	14 (1.8)	3 (0.8)	7 (2.0)	0.205	6 (1.4)	4 (1.3)	1.000
Myocardial infarction (%)	13 (1.7)	4 (1.0)	8 (2.3)	0.245	7 (1.6)	5 (1.6)	1.000
Stroke	4 (0.5)	2 (0.5)	2 (0.6)	1.000	3 (0.7)	1 (0.3)	0.646
Target-vessel revascularisation (%)	8 (1.1)	2 (0.5)	6 (1.7)	0.159	3 (0.7)	5 (1.6)	0.286
Composite of death, MI, stroke, TVR (%)	28 (3.7)	8 (2.0)	16 (4.5)	0.062	15 (3.4)	9 (2.9)	0.834

*NOAC* non-vitamin K antagonist oral anticoagulant, *VKA* vitamin K antagonist, *DT* dual therapy, *TT* triple therapy, *BARC* Bleeding Academic Research Consortium, *TIMI* thrombolysis in myocardial infarction, *ISTH* International Society on Thrombosis and Haemostasis, *CRNM* clinically relevant non-major, *MI* myocardial infarction, *TVR* target-vessel revascularisation

##### Thrombotic events at 30 days

Thrombotic events occurred in 3.7% of patients. More thrombotic events occurred among patients treated with VKAs as compared to NOACs, but this difference was not statistically significant (4.5% vs 2.0%, *p* = 0.06). There were more myocardial infarctions (2.3% vs 1.0%, *p* = 0.25), urgent target-vessel revascularisation (1.7% vs 0.5%, *p* = 0.16) and all-cause death (2.0% vs 0.8%, *p* = 0.21) in patients on VKAs. The difference in thrombotic events between VKAs and NOACs was smaller after excluding patients not eligible for NOACs (4.2% vs 2.1%, *p* = 0.12). Dual therapy showed comparable antithrombotic efficacy to that of triple therapy (3.4% vs 2.9%, *p* = 0.83). Pre-treatment with aspirin or a P2Y12 inhibitor, or peri-procedural UFH use, did not lead to a reduction in thrombotic events (4.2% vs 2.7%; 3.9 vs 2.0%; and 3.7% vs 3.4%). We found a negative correlation with the year of the procedure, indicating fewer thrombotic complications over time.

##### Bleeding at 30 days

Bleeding occurred in 16% of all patients. Patients in whom the OAC therapy was interrupted versus continued (15% vs 16%) and in whom femoral versus radial access (16% vs 15%) was used showed comparable bleeding rates. Bleeding rates were similar in patients on NOACs and those on VKAs (17% vs 14%, *p* = 0.42), also after excluding patients not eligible for NOACs (mechanical heart valve and eGFR < 30). Although the bleeding rate was higher in patients treated with triple therapy than in those on dual therapy (18% vs 14%, *p* = 0.26), the difference did not reach statistical significance.

### Discussion

This multi-centre registry provides insight into the antithrombotic management of patients requiring oral anticoagulation for AF or a mechanical heart valve prosthesis who undergo PCI with stenting, thus requiring additional antiplatelet therapy. We observed that (1) the population consisted of elderly patients with both a high bleeding and ischaemic risk; (2) treatment with NOACs or VKAs and dual or triple therapy was roughly equal, while over time a strong preference for more dual therapy consisting of a NOAC and clopidogrel was seen; (3) patients discharged on VKAs more often had comorbid vascular disease than those discharged on NOACs; (4) patients discharged on dual therapy more frequently had chronic heart failure, anaemia and malignancy as compared to those discharged on triple therapy.

The main findings at 30 days were that: (1) the rate of neither thrombotic nor bleeding events was significantly different between patients on VKAs and those on NOACs; (2) the rate of thrombotic and bleeding events was not significantly different between patients on triple therapy and those on dual therapy.

Among patients who have an indication for oral anticoagulation, about one-third also have coronary artery disease for which, at some time, PCI may be indicated [17]. There is still controversy as to what is the best antithrombotic regime for patients on OACs undergoing PCI. European guidelines currently recommend the use of triple therapy (aspirin, clopidogrel and a NOAC) for at least 1 month in patients with AF who undergo PCI [1, 3, 4]. Depending on the ischaemic and bleeding risk, triple therapy can be prolonged up to 6 months. However, in patients with a high bleeding risk, dual therapy with clopidogrel and a NOAC can be used as an alternative to triple therapy. Attempts have been made to find an antithrombotic regimen that can prevent both stroke and coronary events while minimising the risk of bleeding in patients with AF undergoing PCI. Based on the WOEST RCT and observational studies, dual therapy with clopidogrel and an OAC has been shown to reduce bleeding [8, 9]. However, its efficacy in preventing both stroke and coronary events is less certain, since none of the trials had sufficient power to detect differences in thromboembolic events. Therefore, the current practice guidelines recommend dual therapy as a viable option only in patients with a high bleeding risk [16–18]. In line with these findings, in our analysis the rate of 30-day bleeding was higher only in patients treated with triple therapy compared to dual therapy (18% vs 14%, *p* = 0.26). The antithrombotic efficacy of both treatments was similar. These results probably demonstrate that physicians, aware of both the thrombotic and bleeding risk, are capable of choosing the right combination of drugs, e.g., triple therapy where a high thrombotic risk prevails and dual therapy for those patients with a high bleeding risk.

For patients with AF, NOACs have proved to be at least equally effective as VKAs in preventing stroke or systemic embolism with a similar to superior safety profile in reducing bleeding [12–15]. Recent trials have investigated the safety of dual therapy consisting of a NOAC plus a P2Y12 inhibitor in comparison with triple therapy with a VKA in the setting of patients with AF undergoing PCI or with ACS. The RE-DUAL PCI, PIONEER AF-PCI and ENTRUST-AF PCI trials demonstrated that dual therapy with a NOAC (dabigatran, rivaroxaban and apixaban, respectively) plus a P2Y12 inhibitor, thus omitting aspirin, resulted in reduced bleeding compared to triple therapy with a VKA, without an apparent trade-off for ischaemic events [19–22]. However, the AUGUSTUS trial was the only one to compare treatment with dual therapy with either a VKA or a NOAC among patients with AF and ACS or PCI. Dual therapy with apixaban resulted in less bleeding than dual therapy with a VKA [23]. This has resulted in updated European guidelines and expert consensus, with a shift in preference towards a NOAC over a VKA as part of dual or triple therapy in patients with AF undergoing PCI [18, 24, 25]. This was also observed in our registry as a strong preference for more treatment with NOACs and less triple therapy over the years.

As compared to those studied in the AF-PCI RCTs, our cohort probably has a higher risk owing to: a higher age (mean age 74 vs 70 years, 47% vs 35% above 75 years); more previous CABGs (20% vs 6–10%) and PCIs (36% vs 31–35%); worse renal function (mean eGFR 61 ml/kg per minute versus 72–79 ml/kg per minute); and a higher CHA_2_DS_2_-VAC score (3.9 vs 3.3–3.9) [19–21, 23].

All four PCI-AF RCTs reported less bleeding among patients treated with NOACs than in those on VKAs with similar thrombotic event rates. Only the AUGUSTUS trial reported a significantly lower occurrence of stroke with dual therapy with apixaban [23]. In our analysis, there were more thrombotic events among patients treated with VKAs, but similar bleeding rates between patients on NOACs and VKAs. An explanation for this difference from the PCI-AF RCTs could be the observational nature of the study, resulting in different baseline characteristics between the two groups. The higher thrombotic risk with the use of VKAs might also be partly due to VKAs being associated with a higher plaque burden and increased high-risk plaque features in coronary artery disease [26]. Patients discharged on VKAs in our study had a higher baseline bleeding and thrombotic risk than patients on NOACs. A similar bleeding rate despite a higher bleeding risk in the VKA group might be explained by aspirin being prescribed less frequently. Direct comparison of the occurrence of bleeding and thrombotic events in our registry and the RCTs was not possible, as our analysis reported on 30-day event rates and the trials on 6‑ to 12-month event rates.

To further prevent bleeding, current guidelines recommend peri-procedural measures such as use of a radial approach during PCI, second-generation DES use, avoidance of the use of glycoprotein IIb/IIIa inhibitors and avoidance of peri-procedural bridging with heparin if not strictly indicated [18]. For two-thirds of all patients in our study a radial access site was used, > 90% received second-generation DES, and glycoprotein IIb/IIIa inhibitors were used in only 4%. These results seem to be in line with current recommendations. Furthermore, in our study oral anticoagulation therapy was interrupted before PCI in more than one-third of patients, while current guidelines recommend the use of uninterrupted anticoagulation for elective PCI. However, a meta-analysis found that the rate of bleeding and 30-day major adverse cardiovascular events was similar with interrupted or uninterrupted VKA therapy in AF patients undergoing PCI [27]. This is in line with our findings, as the 30-day rate of bleeding and thrombotic events did not differ regardless of whether anticoagulation therapy was interrupted before PCI or not. Of all the patients discharged on NOACs, 77% received a UFH bolus during PCI, which is in line with current recommendations. Guidelines recommend the use of parenteral anticoagulants during PCI in AF patients on NOACs regardless of the timing of the last NOAC dose [28]. This is based on a small pilot study in 50 stable patients undergoing planned PCI and on DAPT, suggesting that pre-procedural dabigatran provides insufficient anticoagulation during PCI [29]. A similar study with rivaroxaban, however, showed suppressed coagulation activation after elective PCI, without increased bleeding [30].

#### Study strength and limitations

The main strength of this multi-centre registry is the detailed description of a relatively large all-comer population. We were able to describe practice over time and especially of the uptake of NOACs. To the best of our knowledge, our study is the first to provide an in-depth description of baseline and peri-procedural characteristics as well as the drivers for prescribing antithrombotic therapy, including NOACs.

Our study has several limitations. First, the data included in our analysis, although prospective, came from an observational study. Furthermore, we could not compare patients treated with clopidogrel versus stronger P2Y12 inhibitors, as the majority of patients (> 90%) received clopidogrel. There was also a low event rate for bleeding and ischaemic events and our study was not powered to detect differences in event rates between groups. Therefore, the results of our study show only trends and are for hypothesis generation only. Further investigation is needed.

## Supplementary Information

Overview of predictors for different type of antithrombotic strategies and outcome events.
