# Supplementary material for: The WOEST 2 registry: A prospective registry on antithrombotic therapy in atrial fibrillation patients undergoing percutaneous coronary intervention
Source: Neth Heart J. 2022 Mar 1;30(6):302–11. doi: 10.1007/s12471-022-01664-0 (PMC9123099; doi:10.1007/s12471-022-01664-0)
Supplement: Supplementary file 1 — Overview of predictors for different type of antithrombotic strategies and outcome events. [file 12471_2022_1664_MOESM1_ESM.docx]

**SUPPLEMENTARY MATERIAL**

**Table S1a. Predictors for VKA or NOAC prescription.**

| Log reg on VKA (vs NOAC) *correction for year of procedure | | |  |
| --- | --- | --- | --- |
|  | **Beta** | **Odds Ratio** | **p-value** |
| Atrial fibrillation | -1.226 | 0.293 | 0.001 |
| Myocardial infarction | 0.603 | 1.828 | 0.001 |
| CABG | 0.607 | 1.835 | 0.003 |
| Congestive heart failure with LVEF <50% | 0.625 | 1.868 | 0.005 |
| BMS placement | -0.991 | 0.371 | 0.006 |
| PCI | 0.457 | 1.580 | 0.007 |
| Femoral access | -0.389 | 0.678 | 0.026 |
| STEMI at presentention | 0.730 | 2.075 | 0.029 |
| Peripheral artery disease | 0.465 | 1.592 | 0.040 |

*excl gfr<30 en indicatie kleplijden

**Table S1b. Predictors for triply or dual therapy prescription.**

| Log reg on triple (vs dual) *correction for year of procedure | |  |  |
| --- | --- | --- | --- |
|  | **Beta** | **Odds Ratio** | **p-value** |
| Alcohol abuse (>7units/week) | -1.148 | 0.317 | 0.000 |
| Congestive heart failure with LVEF <50% | -0.890 | 0.410 | 0.000 |
| BMS placement | 1.112 | 3.039 | 0.001 |
| Valvular disease | -0.592 | 0.553 | 0.002 |
| Hypertension | 0.460 | 1.584 | 0.005 |
| ACS at presentation | 0.437 | 1.548 | 0.007 |
| Anemia | -0.706 | 0.493 | 0.014 |
| Malignancy | -0.526 | 0.591 | 0.033 |

*excl gfr<30 en indicatie kleplijden

Note: NOAC: non-vitamin K antagonist, VKA: vitamin K antagonist, ACS: acute coronary syndrome, STEMI: ST-elevation myocardial infarction, LVEF: left ventricular ejection fraction, GFR: glomular filtration rate, BMS: bare metal stent.

**SUPPLEMENTAL MATERIAL**

**Table S2. Predictors of outcome events.**

**Table S2a.**

| Log reg on comp thrombotic outcome |  |  |  |
| --- | --- | --- | --- |
|  | **Beta** | **Odds Ratio** | **p-value** |
| ACS at presentation | 1.135 | 3.111 | 0.004 |
| Multivessel disease | 0.940 | 2.559 | 0.022 |
| Stroke | 0.948 | 2.580 | 0.028 |
| CHADS-VASc >3 | 0.234 | 1.264 | 0.047 |
| Age | 0.050 | 1.051 | 0.056 |

**Table S2b.**

| Log reg on bleeding outcome |  |  |  |
| --- | --- | --- | --- |
|  | **Beta** | **Odds Ratio** | **p-value** |
| PCI | -0.697 | 0.498 | 0.003 |
| Peptic ulcer disease | 1.272 | 3.568 | 0.010 |
| Gastro-intestinal bleed | 0.802 | 2.231 | 0.063 |

**Table S2c.**

|  |  | Bleeding events N (%) | | | Thrombotic events N (%) | | |  |
| --- | --- | --- | --- | --- | --- | --- | --- | --- |
| PROCEDURAL | | | | | | | |  |
| Elective | No (215) vs Yes (543) | 39 (18.1) | 80 (14.7) | 0.268 | 15 (7.0) | 13 (2.4) | 0.005 | |
| Interruption of OAC | No (492) vs Yes (243) | 80 (16.3) | 37 (15.2) | 0.749 | 21 (4.3) | 7 (2.9) | 0.418 | |
| Femoral access | No (496) vs Yes (262) | 76 (15.3) | 43 (16.4) | 0.753 | 21 (4.2) | 7 (2.7) | 0.318 | |
| Guiding > 6Fr | No (703) vs Yes (36) | 112 (15.9) | 6 (16.7) | 0.819 | 28 (4.0) | 0 (0.0) | 0.391 | |
| PERIPROCEDURAL ANTITHROMBOTIC THERAPY | | | | | | | |  |
| Oral anticoagulant naive at admission | No (674) vs Yes (84) | 104 (15.4) | 15 (17.9) | 0.528 | 21 (3.1) | 7 (8.3) | 0.027 | |
| Anti-platelet therapy naive at admission | No (308) vs Yes (450) | 35 (11.4) | 84 (18.7) | 0.008 | 11 (3.6) | 17 (3.8) | 1.000 | |
| ASA at admission | No (521) vs Yes (237) | 86 (16.5) | 33 (13.9) | 0.391 | 17 (3.3) | 11 (4.6) | 0.406 | |
| ASA at any moment before PCI | No (263) vs Yes (495) | 38 (14.4) | 81 (16.4) | 0.530 | 7 (2.7) | 21 (4.2) | 0.317 | |
| P2Y12 at admission | No (344) vs Yes (414) | 64 (18.6) | 55 (13.3) | 0.056 | 12 (3.5) | 16 (3.9) | 0.848 | |
| P2Y12 at any moment before PCI | No (98) vs Yes (660) | 17 (17.3) | 102 (15.5) | 0.655 | 2 (2.0) | 26 (3.9) | 0.564 | |
| UFH use | No (116) vs Yes (642) | 16 (13.8) | 103 (16.0) | 0.677 | 4 (3.4) | 24 (3.7) | 1.000 | |
| < 70 units/kg | No (478) vs Yes (131) | 82 (17.2) | 15 (11.5) | 0.138 | 14 (2.9) | 7 (5.3) | 0.182 | |
| >100 units/kg | No (394) vs Yes (215) | 63 (16.0) | 34 (15.8) | 1.000 | 18 (4.6) | 3 (1.4) | 0.060 | |
| GPIIb/IIIa use | No (731) vs Yes (27) | 118 (16.1) | 1 (3.7) | 0.104 | 26 (3.6) | 2 (7.4) | 0.263 | |
| ANTITHROMBOTIC THERAPY AT DISCHARGE | | | | | | | |  |
| Antithrombotic strategy at discharge | Dual (437) vs Triple (309) | 63 (14.4) | 54 (17.5) | 0.263 | 15 (3.4) | 9 (2.9) | 0.834 | |
| * VKA | VKA dual (225) vs VKA triple (128) | 28 (12.4) | 23 (18.0) | 0.160 | 10 (4.4) | 6 (4.7) | 1.000 | |
| * NOAC triple (vs dual) | NOAC Dual (212) vs NOAC triple (181) | 35 (16.5) | 31 (17.1) | 0.893 | 5 (2.4) | 3 (1.7) | 0.731 | |
| Type of OAC at discharge  *excl egfr<30 en metallic valve | NOAC (393) vs VKA (261) | 66 (16.8) | 36 (13.8) | 0.323 | 8 (2.0) | 10 (3.8) | 0.222 | |
| Type of P2Y12 at discharge | Clopidogrel (707) vs ticagrelor/prasugrel (46) | 113 (16.0) | 5 (10.9) | 0.528 | 24 (3.4) | 2 (4.3) | 0.669 | |

Note: OAC: oral anticoagulation, ASA: aspirin, PCI: percutaneous coronary intervention, UFH: unfractionated heparin, NOAC: non-vitamin K antagonist, VKA: vitamin K antagonist, ACS: acute coronary syndrome.
